# The Effects of Natural Product-Derived Extracts for Longitudinal Bone Growth: An Overview of In Vivo Experiments

**DOI:** 10.3390/ijms242316608

**Published:** 2023-11-22

**Authors:** Dong Wook Lim, Changho Lee

**Affiliations:** Division of Functional Food Research, Korea Food Research Institute, Wanju 55365, Republic of Korea; dwlim@kfri.re.kr

**Keywords:** longitudinal bone growth, idiopathic short stature, growth plate, chondrogenesis, natural products

## Abstract

Approximately 80% of children with short stature are classified as having Idiopathic Short Stature (ISS). While growth hormone (GH) treatment received FDA approval in the United States in 2003, its long-term impact on final height remains debated. Other treatments, like aromatase inhibitors, metformin, and insulin-like growth factor-1 (IGF-1), have been explored, but there is no established standard treatment for ISS. In South Korea and other Asian countries, East Asian Traditional Medicine (EATM) is sometimes employed by parents to potentially enhance their children’s height growth, often involving herbal medicines. One such product, *Astragalus membranaceus* extract mixture HT042, claims to promote height growth in children and has gained approval from the Korean Food and Drug Administration (KFDA). Research suggests that HT042 supplementation can increase height growth in children without skeletal maturation, possibly by elevating serum IGF-1 and IGF-binding protein-3 levels. Preclinical studies also indicate the potential benefits of natural products, including of EATM therapies for ISS. The purpose of this review is to offer an overview of bone growth factors related to ISS and to investigate the potential of natural products, including herbal preparations, as alternative treatments for managing ISS symptoms, based on their known efficacy in in vivo studies.

## 1. Introduction

Idiopathic short stature (ISS) is defined as an individual’s height below the 3% relative to the average height for the given age without finding any specific environmental, nutritional or genetic abnormality [1]. Approximately 80% of children with short stature are presented as having ISS [2]. The treatment with recombinant human growth hormone (rhGH) for ISS was approved by the Food and Drug Administration (FDA) in the United States in July 2003, and the current FDA-approved doses for GH in ISS are up to 0.30–0.37 mg/kg per week [3]. rhGH treatment increases the growth rate in the first year, but its effect on final height for 5 years is controversial [4]. Given that it requires daily parenteral administration, and its cost remains prohibitive in many parts of the world, a new strategic approach is needed [5]. Recently, efforts have been made to explore therapeutic alternatives that would facilitate slower bone maturation and more prolonged spontaneous or induced growth. Treatment options, such as aromatase inhibitors, gonadotropin-releasing hormone analogues (GnRHas), and recombinant human insulin-like growth factor-1 (rhIGF-1), have been employed to address ISS [6]. Several studies have shown that GnRHas can enhance the final adult height in ISS children with central precocious puberty, with the most significant effect observed in girls under 10 years of age [7]. Indeed, the combination of GnRHas and rhGH has seen an increased use in the treatment of ISS with precocious puberty [8]. However, at present, there is no standard treatment, and the management of ISS remains controversial [6].

In Asian countries, including South Korea, parents have used East Asian Traditional Medicine (EATM) treatments to potentially promote height growth in their children. ISS is one of the most common symptoms encountered by children and adolescents seeking alternative therapies at oriental medicine hospitals, and several kinds of herbal medicines have been administered as alternative treatments in South Korea [9]. Although the scientifically clear efficacy of helping ISS is controversial, various prescriptions of herbal medicines are used for ISS symptoms in South Korea [10]. The *Astragalus* extract mixture HT042 is a standardized functional food ingredient approved by the Korean Food and Drug Administration (KFDA, 2014) with the claim that ‘HT042 can help height growth of children’. It has been reported that the supplementation of HT042 for 23 weeks increased the height growth in children without skeletal maturation, and this effect was attributed to elevated serum IGF-1 and IGF-binding protein-3 levels [11]. Indeed, several preclinical studies have indicated that the herbal decoction of EATM has potential benefits for the treatment of ISS [12]. Therefore, although efficacy may be controversial, natural products, including herbal preparations, may offer a potential alternative medicine for managing short stature. In this review, we aim to summarize our present knowledge of the longitudinal bone growth factors contributing to the pathogenesis of ISS. Additionally, we explore the potential of natural products in promoting bone length growth through the use of animal models and key biomarkers.

## 2. The Growth Hormone (GH)–Insulin-like Growth Factor (IGF) Axis

**Figure 1:**
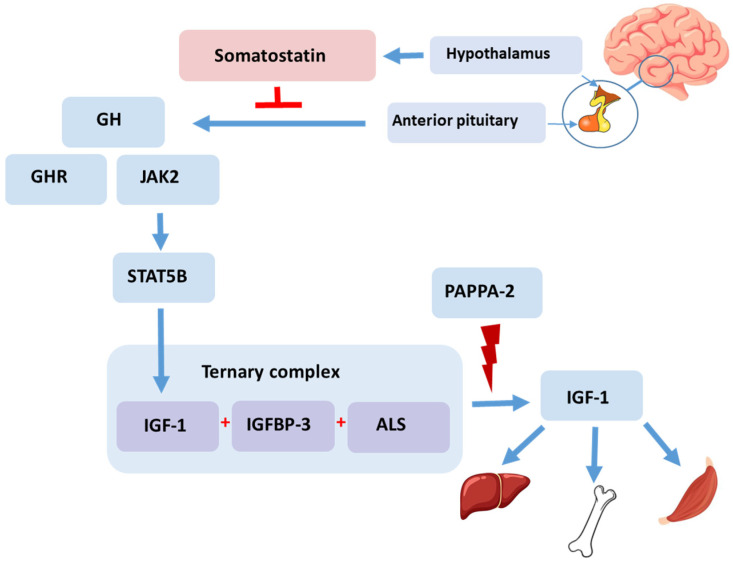
Growth hormone–insulin-like growth factor-1 axis. GH binds to a GHR, which activates JAK2 and promotes the phosphorylation of various members of the STAT5B protein family. This process primarily stimulates the production of IGF-1. IGF-1 circulates in the bloodstream, primarily bound to IGFBP-3 and ALS, forming a ternary complex. PAPPA-2 proteolyzes IGFBP-3, increasing the bioavailability of IGF-1. As the ternary complex reaches the growth plate, it interacts with specific IGF-1 receptors located on the surface of chondrocytes, which are responsible for bone growth. GH: growth hormone, GHR: growth hormone receptor, JAK2: Janus kinase 2, STAT5B: signal transducer and activator of transcription 5B, IGF-1: insulin-like-growth factor 1, IGFBP-3: IGF-binding protein-3, ALS: acid-labile subunit, PAPPA-2: Pregnancy-associated plasma protein-2.

Although ISS does not present with any specific environmental, nutritional, or genetic abnormality, it is necessary to approach it from the perspective of genetic defects in the somatotropic axis. The somatotropic axis, also known as the GH–IGF axis, is composed of GH, IGFs, related carrier proteins, and receptors (Figure 1). It plays an important role in regulating metabolic and physiological processes, including bone growth [13]. Some patients with ISS show abnormalities in the somatotropic axis, indicating GH insensitivity [14]. GH is primarily regulated by the interaction of two peptide hormones: GH-releasing hormone, which stimulates the secretion of growth hormone, and somatostatin, which inhibits GH secretion from the hypothalamus [15]. GH binds to a GH receptor (GHR), which activates JAK2 (Janus Kinase 2) and promotes the phosphorylation of various members of the signal transducer and activator of transcription family (STAT5B). This process primarily stimulates IGF-1. IGF-1, a single-chain polypeptide, is a growth-promoting polypeptide that is essential for normal growth and development [16]. IGF-1 is closely related to the GH level and plays a crucial role in promoting the growth and development of bones, particularly in the growth plates [17]. It has been reported that children with ISS might exhibit lower IGF-1 levels compared to the average values observed in healthy children [18]. The growth plate, also known as the epiphyseal plate, is a specialized cartilaginous region at the ends of long bones where bone growth occurs during childhood and adolescence [19]. IGF-1 circulates in the bloodstream, primarily bound to IGF-binding protein-3 (IGFBP-3) and acid-labile subunit (ALS), forming a ternary complex (IGF-1/IGFBP-3/ALS) [20]. As the ternary complex reaches the growth plate, it interacts with specific IGF-1 receptors located on the surface of chondrocytes, which are the cells responsible for bone growth [21]. Pregnancy-associated plasma protein-2 (PAPPA-2) is a metalloproteinase enzyme that plays a role in cleaving IGFBP. Specifically, it proteolyzes IGFBP-3, leading to an increase in the bioavailability of IGF-1 [22].

## 3. Regulator of Growth Plate Chondrogenesis

**Figure 2:**
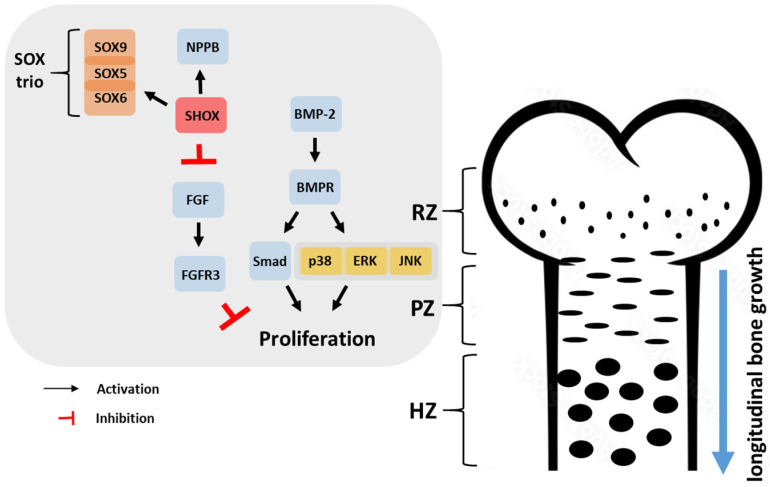
Bone growth and mechanism of FGF and SHOX on growth plate chondrogenesis. The RZ stores the precursor cells supporting growth plate maintenance and bone growth. The PZ is crucial for active cell replication in endochondral bone formation. The HZ contains large, non-dividing chondrocytes contributing significantly to overall growth. SHOX stimulates chondrocyte proliferation and differentiation by activating NPPB, inhibiting FGFR3 expression, and interacting with the SOX trio (SOX9, SOX5, and SOX6) for cartilage matrix production. FGFR3 negatively impacts growth plate chondrogenesis. RZ: Resting zone, PZ: Proliferative zone, HZ: Hypertrophic zone, BMP-2: Bone morphogenetic protein-2, BMPR: Bone morphogenetic protein receptor, p38: p38 MAP kinase, ERK: extracellular signal-regulated kinase, JNK: c-Jun NH2-terminal kinase, NPPB: Natriuretic peptide precursor B gene, SHOX: Short stature homeobox, FGF: Fibroblast growth factor, FGFR3: Fibroblast growth factor receptor 3.

The growth plate, comprising the resting zone (RZ), proliferative zone (PZ), and hypertrophic zone (HZ), is a crucial structure involved in bone development [23]. According to previous reports on the mechanistic pathways of human height growth [24], chondrocytes in the RZ are irregularly distributed within the cartilage matrix layer, while those in the PZ and HZ align in columns parallel to the bone’s long axis. The RZ acts as a reservoir of precursor cells that support growth plate maintenance and contribute to bone growth. The PZ plays a crucial role in endochondral bone formation as it hosts active cell replication. Similarly, the HZ consists of chondrocytes derived from the terminal differentiation of PZ chondrocytes located the farthest from the epiphysis. These hypertrophic chondrocytes cease dividing and undergo significant enlargement, making substantial contributions to the overall growth process (Figure 2).

Bone morphogenetic protein-2 (BMP-2), known for its potent ability to induce osteogenic differentiation, binds to its BMP receptor (BMPR) and activates the Smad signaling (canonical signaling) pathway, thereby stimulating bone formation [25]. In addition to Smad signaling (canonical signaling), the mitogen-activated protein kinase (MAPK) cascade, which represents a non-canonical pathway for BMP-2, activates p38 MAP kinase, extracellular signal-regulated kinase (ERK), and c-Jun NH 2-terminal kinase (JNK), leading to the osteogenesis-specific promotion of bone formation through the activation of runt-related transcription factor 2 (Runx2) transcription factor [26]. Recently, the growth plate has gained increasing importance in the study of the genetic causes of short stature. Fibroblast growth factors (FGFs) play a crucial role in the growth plate. Among these factors, the fibroblast growth factor receptor-3 (FGFR3) negatively regulates growth plate chondrogenesis [27]. The activation of FGFR3 stimulates various pathways, including Signal transducer and activator of transcription 1 (STAT1), mitogen-activated protein kinase (MAPK), and protein phosphatase 2a (PP2a), which in turn trigger downstream signals (p107, p21Waf1/Cip1, and Sox9) [28]. This leads to the suppression of chondrocyte proliferation while also regulating matrix production and chondrocyte differentiation. Another significant factor is Short stature homeobox (SHOX), a transcriptional activator expressed in hypertrophic chondrocytes. SHOX plays a role in stimulating and coordinating chondrocyte proliferation and differentiation. It achieves this by activating natriuretic peptide precursor B gene (NPPB) and inhibiting FGFR3 expression, thereby promoting longitudinal growth. Additionally, SHOX interacts with the SOX trio (SOX9, SOX5, and SOX6 genes), which are involved in cartilage matrix production. Defects in the SHOX gene impact approximately 2–15% of children classified as ISS, with the prevalence varying based on the study and the cohort of children examined [29]. Indeed, genome-wide association (GWA) studies suggested that the major effect on height variability is due to rare variants in the genes involved in growth plate development [30].

## 4. Animal Models for Studying the Effect of Longitudinal Bone Growth

Hypophysectomy was performed in rats to establish an animal model of growth hormone deficiency [31]. Evidence from animal models shows that hypophysectomy in rats significantly decreased body weight, tail length, and bone lengths, as well as bone mineral density. These results improved to normal levels with the treatment of growth hormone through daily injections of 0.25 mg/kg of rhGH [32]. However, hypophysectomy also renders the animals deficient in other growth-regulating hormone systems. Therefore, GH deficiency produced by hypophysectomy may not be the ideal model to study the growth effects of hormone replacement strategies [33]. As summarized in the Table in Section 5.20, to assess the impact of the extract on bone length growth, mainly predominantly adolescent Sprague–Dawley (SD) or Wistar rats aged 3 to 4 weeks, specifically targeting the stage before the adolescent phase [34], were used. Most extracts were administered orally, with recombinant human growth hormone (rhGH) used as a positive control. During the in vivo experiment, changes in body weight and lengths from the nose to the tail or anus in response to extract administration were assessed. After sacrifice, changes in the lengths of the growth plate were measured following tetracycline labeling using fluorescence microscopy [35]. It also determined the levels of proteins associated with bone growth-related biomarkers, such as IGF-1, BMP-2, and IGFBP-3, in both serum and tissue-sectioned growth plates. Additionally, bone mineral contents (BMCs) and bone mineral density (BMD) were assessed using dual energy X-ray absorptiometry (DEXA) or micro-CT equipment.

## 5. Natural Products-Derived Extracts, including Herbal Formulations, for Short Stature

**Figure 3:**
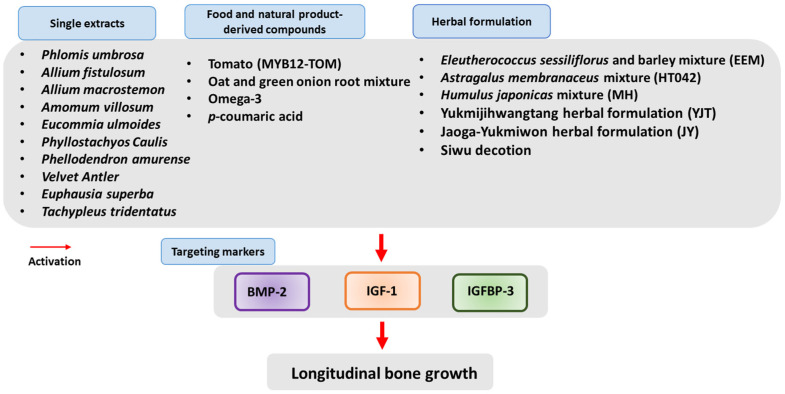
Effect of natural products, including of herbal formulations, on longitudinal bone growth by the activation of growth hormone-mediated biomarkers in animal models. Natural products may enhance longitudinal bone growth by regulating BMP-2, IGF-1, and IGFBP-3. IGF-1: Insulin-like-growth factor 1, IGFBP-3: IGF-binding protein-3, BMP-2: Bone morphogenetic protein-2.

Multiple studies have demonstrated that changes in bone growth-related biomarkers, such as IGF-1, BMP-2, and IGFBP-3, contribute significantly to the development of longitudinal bone growth. Therefore, targeting these markers through the regulation of growth hormone has been proposed as a therapeutic strategy for the treatment of ISS [36]. Many natural products, including herbal formulations, have been found to exert enhancing effects on longitudinal bone growth in animal models by increasing IGF-1, BMP-2, and IGFBP-3 levels in both the serum and tissue-sectioned growth plates. These studies suggest that natural products may enhance longitudinal bone growth by regulating IGF-1. Figure 3 and Table 1 present a summary of the natural products that have been found to possess the potential to alleviate longitudinal bone growth.

### 5.1. Eleutherococcus sessiliflorus and Barley Mixture (EEM)

*E. sessiliflorus* is a medicinal herb with a range of biological effects, including anti-inflammatory, anti-cancer, antioxidant, and anti-obesity properties [37]. Barley (*Hordeum vulgare*) stands out as one of the most crucial crops globally. Barley has been demonstrated to effectively remove superoxide anion radicals [38]. In vivo experiments have confirmed that EEM, a standardized mixture of these two components, *E. sessiliflorus* and barley, promotes bone length growth in rats [39]. Briefly, the length of the proximal tibial growth plate was increased by the oral administration of EEM 50 mg/kg and 200 mg/kg for 7 days in female adolescent SD rats. Indeed, the administration of EEM markedly increased the intensity of IGF-1 expressions in the proliferative and hypertrophic zones of growth plate. Also, EEM was significantly increased in liver IGF-1 and IGFBP-3 mRNA expressions [39].

### 5.2. Astragalus membranaceus Mixture (HT042)

HT042 is a mixed herbal formulation comprising the dried roots of *Phlomis umbrosa*, the roots of *Astragalus membranaceus*, and the stem barks of *Eleutherococcus senticosus* [40]. The group treated with HT042 at a dosage of 100 mg/kg for four days showed a significant increase in the proximal tibia growth plate of female SD rats. Additionally, the number of 5-bromodeoxyuridine (BrdU)-positive cells in the HT042 group was significantly higher compared to that of the control group in the tibia growth plate. Moreover, the expression levels of BMP-2 and IGF-1 were notably elevated in the PZ and HZ of growth plate in the HT042-treated group. The randomized controlled trial demonstrated that supplementation with HT042 for 23 weeks increased height growth in children who had not yet reached skeletal maturation [11]. This effect was attributed to elevated levels of serum IGF-1 and IGF-binding protein-3 [41]. HT042 has been recognized as a standardized functional food ingredient approved by the KFDA, 2014, with the statement that ‘May support physical growth of young children’.

### 5.3. Humulus japonicus Mixture (MH)

*H. japonicus*, commonly known as Japanese hops, is used for the treatment of inflammatory diseases [42]. In an in vivo study, it was observed that nose–tail length gain, body weight gain, and femur and tibia length significantly increased in the group treated with *H. japonicus* water extracts combined with garlic and watermelon powder (MH) at dosages of 100 and 300 mg/kg for 5 weeks in adolescent female SD rats. Additionally, the bone mineral density of the trabecular bone was significantly increased in the MH-treated group, especially at a dosage of 300 mg/kg [43]. These effects may be attributed to the enhancement in IGF-1 and IGFBP-3 levels via the Janus kinase 2 (JAK2)/signal transducer and activator of transcription 5 (STAT5) signaling pathway in the liver [44], as described in Figure 1.

### 5.4. Yukmijihwang-Tang (YJT) Herbal Formulation

Yukmijihwang-tang (YJT) contains six medicinal herbs, such as *Rehmannia glutinosa*, *Cornus officinalis*, *Dioscorea batatas*, *Alisma orientale*, *Poria cocos*, and *Paeonia suffruticosa*, in traditional Korean medicine (TKM) [45]. YJT is widely used in TKM to treat age-related disorders, such as Alzheimer’s disease [46]. An in vivo study showed that YJT, at a dosage of 300 mg/kg in the treated group, significantly increased the length of the proximal tibia in the growth plate of female SD rats. Moreover, YJT stimulated the expression of IGF-1 and BMP-2 in the proximal tibial growth plate [47]. A survey study showed that some oriental medicine clinics primarily use YJT for promoting height growth in children [48], but there is a lack of scientific clinical evidence to support this.

### 5.5. Jaoga-Yukmiwon (JY) Herbal Formulation

Jaoga-Yukmiwon (JY) consists of the alcohol extracts of seven medicinal herbs: *Acanthopanax senticosus*, *Rehmannia glutinosa*, *Poria cocos*, *Dioscorea japonica*, *Cornus officinalis*, *Cervus Nippon*, and *Panax ginseng*. JY is an herbal formulation from TKM used for the treatment of growth disorders [10]. The administration of JY at a dosage of 100 mg/kg showed a significant increase in the tibial length and the induction of BMP-2 in the tibial growth plates in adolescent male SD rats [49].

### 5.6. Siwu Decoction

Siwu decoction, consisting of *Angelica sinensis*, *Cnidium officinale*, *Paeonia lactiflora*, and steam-prepared root of *Rehmannia glutinosa*, has traditional uses for pain relief, anemia improvement, and blood circulation. The main ingredients responsible for these effects are quercetin, kaempferol, cytoglucide, apigenin, and stigmasterol [50]. Administered as water extracts to 33-day-old SD rats twice daily for 4 days, it promoted cartilage cell proliferation and new bone formation in the growth plate. Additionally, in the proximal tibial growth plate, the Siwo decoction increased IGF-1 and BMP-2 expressions [51].

### 5.7. Phlomis umbrosa

The *P. umbrosa* root has been used in traditional Chinese medicine to enhance muscular and skeletal strength and to address fractures. In previous reports, *P. umbrosa* was found to possess various properties, including anti-allergic, anti-inflammatory, and anti-nociceptive effects [52]. These effects are likely attributed to the presence of various active compounds, such as sesamoside, shanzhiside methyl ester, chlorogenic acid, and barlerin [53]. When administered orally at doses of 100 and 300 mg/kg for 10 consecutive days, *P. umbrosa* root 70% ethanol extracts markedly increased longitudinal bone growth within the proximal tibial growth plate in adolescent SD female rats (5-weeks-old), consistent with findings showing a significant increase in the number of BrdU-labeled chondrocytes in the *P. umbrosa* groups at doses of 100 and 300 mg/kg. Moreover, *P. umbrosa* root extracts elevated serum IGFBP-3 levels and stimulated the expressions of IGF-1 and BMP-2 in the proximal tibial growth plate. These animal study results were similar to the rhGH-treated groups (200 μg/kg, s.c.) used as the positive control [54].

### 5.8. Allium fistulosum

*A. fistulosum* is a perennial plant species that grows globally and belongs to the Liliaceae family. Previous studies reported the effects of *A. fistulosum* on antioxidant activity [55]. Administering 450 mg/kg of *A. fistulosum* water extracts significantly increased the length of the proximal tibial growth plate and bone mineral density in C57BL/6 mice (4-weeks-old) that were fed a diet deficient in vitamin D and calcium for 5 weeks. The in vivo serum analysis demonstrated that *A. fistulosum* extracts elevated bone formation markers, such as alkaline phosphatase, and exhibited the potential to stimulate bone growth through mechanisms involving calcium, osteocalcin, and collagen type 1 [56].

### 5.9. Allium macrostemon

*A. macrostemon*, as an important medicinal and edible herb, has been used to treat some diseases for thousands of years in China [57]. Steroidal saponins, a class of oligoglycosides, are the major active compounds in *A. macrostemon*, and they have biological activities, including hypoglycemic, antithrombotic, and anti-inflammatory effects [58]. The administration of *A. macrostemon* 30% ethanol extracts at a dosage of 100 mg/kg for 10 days significantly increased the proximal tibial length of female SD rats. Additionally, the levels of IGF-1 and BMP-2 were significantly increased in the growth plate of the *A. macrostemon*-treated group [59].

### 5.10. Amomum villosum

*A. villosum* has been traditionally used to improve gastrointestinal motility in traditional medicine [60]. When administered at a dosage of 500 mg/kg for 5 days, *A. villosum* extracts significantly increased the length of the proximal tibial growth plate in female SD rats [61]. BrdU-labeled cells were also significantly observed in the chondrocytes treated with *A. villosum* extracts. Furthermore, the expression levels of BMP-2 and IGF-1 were notably elevated in the pre-hypertrophic and hypertrophic zones of the *A. villosum* extracts-treated group. These effects were similar to those observed in the group treated with rhGH.

### 5.11. Eucommia ulmoides

*E. ulmoides* is well-known as a tonic medicinal herb for the treatment of bone diseases in traditional Chinese medicine [62]. Geniposidic acid, geniposide, and aucubin from *E. ulmoides* have been shown to increase bone metabolism [63]. In the group treated with *E. ulmoides* 70% ethanol extracts at a dose of 100 mg/kg for 4 days, there was a significant enhancement in the length of the proximal tibial growth plate due to the promotion of chondrogenesis in the growth plate, along with increased levels of BMP-2 and IGF-1 in adolescent female SD rats [64].

### 5.12. Phyllostachyos caulis

*P. caulis*, well-known as the stem of giant timber bamboo, is used for the treatment of hypertension and cardiovascular diseases in traditional Chinese medicine [65]. It has various biological effects, such as those related to inflammatory diseases [66]. When administered at a concentration of 200 mg/kg of bamboo extract for 10 days to female SD rats, there was a significant increase in the total tibial length, which was attributed to the heights of the proliferative and hypertrophic zones in the tibia. Additionally, the ratio of BrdU-positive to total cells also increased. Furthermore, the levels of serum osteocalcin, GH, and IGF-1 were significantly elevated in the group treated with bamboo extract at a dosage of 200 mg/kg [67].

### 5.13. Phellodendron amurense

*P. amurense* is one of the major plants in traditional Chinese medicine. It has been used traditionally in folk medicine for hepatitis, dysentery, and gynaecological inflammation [68]. *P. amurense* contains mainly isoquinoline alkaloids, such as berberine chloride [69]. When administered at dosages of 100 and 300 mg/kg for 41 days, *P. amurense* extracts significantly increased the length of the proximal tibial growth plate in female SD rats without changes in food intake or body weights. The levels of IGF-1 and BMP-2 in the proliferative zones of the tibial growth plate significantly increased in the *P. amurense* extract-treated groups. Moreover, *P. amurense* had no significant effects on vaginal opening, ovarian weights, and uterine weights [70].

### 5.14. Velvet Antler

Velvet antler (VA), sourced from young male deer antlers, has a long history of use in Asian countries for its immune-boosting, stamina-enhancing, and bone health benefits. In a study on male SD rats (3-week-old), a 100 mg/kg dose of VA for 5 days significantly increased bone density, growth plate height, and BMP-2 expression. When applied to MG-63 cells, VA also stimulated cell proliferation, ALP activity, collagen synthesis, calcium deposition, and increased mRNA levels of collagen, ALP, and osteocalcin [71].

### 5.15. Euphausia superba

Antarctic krill (*E. superba*) is the largest source of animal protein, and its protein contains all eight essential amino acids, making it a valuable source of potential future food [72]. The peptides from *E. superba*, (AKPs) significantly increased longitudinal bone growth and improved bone strength, contributing to the promotion of chondrocyte proliferation and hypertrophy in the growth plate. This was achieved through the elevation of serum GH and IGF-1 levels, activating the GH/IGF-1 axis signaling pathways. Additionally, AKPs induced the secretion and expression of BMP-2 [73].

### 5.16. Tachypleus tridentatus

*T. tridentatus*, a marine animal used in traditional Chinese medicine, is known for its anti-inflammatory, analgesic, hemostatic, and anti-diarrheal properties. When the plasma of *T. tridentatus* was administered to the female Wistar rats (4-week-old), it exhibited a positive correlation with the rat’s body weight, body length, and tail length. Furthermore, it promoted the growth of the femur and tibia, along with the expansion of the growth plate. Additionally, at a dosage of 200 mg/kg, *T. tridentatus* plasma increased serum levels of ALP, BMP-2, and IGF-1 in rats. Additionally, it significantly upregulated mRNA expression levels of IGF-1 and IGFBP-3 [74].

### 5.17. Omega-3

Omega-3 fatty acids (FAs) are crucial dietary elements that need to be acquired from food sources [75]. It was shown that exposure to high concentrations of omega-3 FAs (EPA, α-linolenic acid, arachidonic acid, and linoleic acid) for 16 weeks accelerates bone growth through alterations of the growth plate, associated with increased chondrocyte proliferation and differentiation in a fat-1 transgenic female mice (3-week-old) [76].

### 5.18. p-Coumaric Acid

Naturally occurring plant phenolic acids may have a beneficial impact on the skeletal system [77]. Among these phenolic acids, *p*-coumaric acid stands out due to its effective inhibition of bone resorption and promotion of bone formation [78]. In a study involving 3-week-old male SD rats, the oral administration of a 100 mg/kg dose of *p*-coumaric acid for 10 days resulted in a significant increase in tibial length and the height of each growth plate zone. Moreover, there was a notable elevation in the expression of IGF-1 and its receptor within the proliferative and hypertrophic zones, along with increased serum levels of growth hormone and IGF-1 in the group treated with *p*-coumaric acid [79].

### 5.19. Oat and Green Onion Root Water Extracts

Orally administered oat and green onion water extracts (GOO) at doses of 100 and 300 mg/kg for 10 consecutive days significantly increased longitudinal bone growth in the tibia and femur length by promoting the proliferation of the growth plates in adolescent SD male rats (3-weeks-old). Furthermore, GOO elevated the secretion of the growth hormone [80].

### 5.20. Tomato (MYB12-TOM)

MYB12-TOM, a genetically engineered flavonol-enriched tomato fruit, was enhanced more than 5-fold compared to wildtype tomato. In a study on female BALB/c mice (3-weeks-old), a 100 mg/kg dose of MYB12-TOM for 6 weeks significantly increased tibial growth and BMP-2 expression for hypertrophic cells compared to the vehicle and wildtype tomato [81].

**Table 1:** Natural product-derived extracts, including herbal formulations, for longitudinal bone growth.

Source	Animals	Analysis	Effective Dose	Components	Standardized Compounds	Ref.
*Eleutherococcus sessiliflorus* and barley mixture (EEM)	Female SD rat (5-weeks-old)	Proximal tibial length IGF-1 BMP-2 IGFBP-3	200 mg/kg for 10 days	*Eleutherococcus sessiliflorus* Barley (*Hordeum vulgare*)	Eleutheroside E Tricin	[39]
*Astragalus membranaceus* mixture (HT042)	Female SD rat (3-weeks-old)	Body weight Nose–tail and anus lengths Proximal tibial length IGF-1 BMP-2	100 mg/kg for 4 days	*Phlomis umbrosa* *Astragalus membranaceus* *Eleutherococcus sessiliflorus*	Chlorogenic acid Caffeic acid Eleutheroside E Ferulic acid Formononetin Shanzhiside methylester	[40]
*Humulus japonicus* mixture (MH)	Female SD rat (3-weeks-old) Hypophysectomized male and female SD rats (3-weeks-old)	Proximal tibial length IGF-1 IGFBP-3 JAK2 STAT5	100 mg/kg for 5 weeks	*Humulus japonicas* Garlic and watermelon powder	-	[43,44]
Yukmijihwangtang herbal formulation (YJT)	Female SD rat (4-weeks-old)	Proximal tibial length IGF-1 BMP-2 IGFBP-3	100 mg/kg for 4 days	*Rehmannia glutinosa* *Cornus officinalis* *Dioscorea batatas* *Alisma orientale* *Poria cocos* *Paeonia suffruticosa*	5-hydroxymethyl-2-furaldehyde Paeoniflorin	[47]
Jaoga-Yukmiwon (JY) herbal formulation	Male SD rat (3-weeks-old)	Proximal tibial length BMP-2	100 mg/kg for 5 days	*Acanthopanax senticosus* *Rehmannia glutinosa* *Poria cocos* *Dioscorea japonica* *Cornus officinalis* *Cervus nippon* *Panax ginseng*	-	[49]
Siwu decotion	Female SD rat (4-weeks-old)	Proximal tibial length IGF-1 BMP-2	100 and 300 mg/kg for 4 days	*Angelica sinensis* *Cnidium officinale* *Paeonia lactiflora* *Rehmannia glutinosa*	Ligustilide 5-hydroxymethyl-2-furaldahyde Paeoniflorin	[51]
*Phlomis umbrosa*	Female SD rat (3-weeks-old)	Proximal tibial length IGF-1 BMP-2 IGFBP-3	1000 mg/kg for 10 days	-	Shanzhiside methyl ester	[54]
*Allium fistulosum*	C57BL/6 mice (4-weeks-old)	Proximal tibial length BMD and BMC Serum ALP, OC, and pro-collagen I alpha 1, calcium	100 mg/kg for 4 weeks	-	N-trans-coumaroyl tyramine N-trans-feruloyltyramine N-trans-feruloyl-3′-methoxytyramine N-trans-decursidate	[56]
*Allium macrostemon*	Female SD rat (3-weeks-old)	Body weight Food intake Proximal tibial length IGF-1 BMP-2	100 mg/kg for 10 days	-	-	[59]
*Amomum villosum*	Female SD rat (4-weeks-old)	Body weight Proximal tibial length IGF-1 BMP-2	500 mg/kg for 4 days	-	-	[61]
*Eucommia ulmoides*	Female SD rat (4-weeks-old)	Proximal tibial length IGF-1 BMP-2	100 mg/kg for 4 days	-	Geniposide	[64]
*Phyllostachyos Caulis*	Male SD rat (3-weeks-old)	Proximal tibial length IGF-1 osteocalcin	200 mg/kg for 4 days	-	-	[67]
*Phellodendron amurense*	Female SD rat (3-weeks-old)	Proximal tibial length IGF-1 BMP-2	300 mg/kg for 41 days		Berberine chloride	[70]
Velvet Antler	Male SD rat (3-weeks-old)	Proximal tibial length BMP-2 ALP activity, collagen synthesis, calcium, osteocalcin	100 mg/kg for 5days	-	-	[71]
*Euphausia superba*	Male ICR mice (3-weeks-old)	Proximal tibial length GH IGF-1 BMP-2	300 mg/kg for 21 days	-	-	[73]
*Tachypleus tridentatus*	Female Wistar rats (4-week-old)	Body weight, body length, and tail length IGF-1 BMP-2	200 mg/kg for 14 days	-	-	[74]
Omega-3	Fat-1 transgenic female mice (3-week-old)	Proximal tibial length	-	-	-	[76]
*p*-coumaric acid	Male SD rats (3-week-old)	Proximal tibial length Growth hormone IGF-1	100 mg/kg for 10 days	-	-	[79]
Oat and green onion root water extracts	Male SD rats (3-week-old)	Proximal tibial length Growth hormone	300 mg/kg for 10 days	-	-	[80]
Tomato (MYB12-TOM)	Female BALB/c mice (3-week-old)	Proximal tibial length BMP-2	100 mg/kg for 6 weeks	-	-	[81]

## 6. Methods

Reference lists were searched for studies published up to 20 September 2023, using the keywords “longitudinal bone growth”, “growth plate”, “idiopathic short stature”, “chondrogenesis”, and “natural products”. When searching these reviews, we did not apply any restrictions on the origin of the material or study design. We excluded articles published in languages other than English.

## 7. Conclusions

The effectiveness of the long-term administration of therapeutic agents used for idiopathic short stature (ISS), such as recombinant human growth hormone (rhGH), aromatase inhibitors, metformin, and insulin-like growth factor-1 (IGF-1), remains a topic of controversy. Consequently, there is an expectation of positive outcomes from easily accessible traditional herbal medicine complexes or health supplements. However, there is a lack of scientific evidence regarding the clear mechanisms of their effects or their potential toxicity. In Korea, only the *Astragalus* extract mixture HT042 has been approved by the Korea Food and Drug Administration (KFDA) to promote height growth in children. Several herbal formulations based on traditional medicine have the potential to improve bone growth, although their effectiveness is controversial. Ongoing research efforts are actively working to uncover a clear mechanism, emphasizing the importance of a scientific approach. Most animal experiments primarily focus on changes in growth hormone, IGF-1, IGFBP-3, and BMP-2 markers. Since GWA studies suggested that the major effect on height variability is due to rare variants in genes involved in growth plate development, it is necessary to conduct research on various biomarkers, such as the activation of FGFs or SHOX, which stimulate various pathways, including the signal transducer in growth plate. As it can be seen in the summarized research results in Table 1, it was confirmed that the majority of extracts containing herbal formulations were orally administered over a short period, ranging from 4 to 10 days during the experimental phase. These short-term studies show clinical limitations in which the effects of bone age maturation cannot be expected. Consequently, it is crucial to assess the efficacy of bone length growth following prolonged administration. Ensuring the safety of long-term extract administration is also of utmost importance. We believe that this mini-review provides a foundational overview for investigating the efficacy of these treatments. Indeed, although toxicological and pharmacological studies are required to determine their safety in humans, natural products, including herbal medicines, are potential therapeutic candidates for the treatment of short stature.

## Data Availability

Not applicable.

## References

[1] Inzaghi E., Reiter E., Cianfarani S. (2019). The Challenge of Defining and Investigating the Causes of Idiopathic Short Stature and Finding an Effective Therapy. Horm. Res. Paediatr..

[2] Derraik J.G.B., Miles H.L., Chiavaroli V., Hofman P.L., Cutfield W.S. (2019). Idiopathic short stature and growth hormone sensitivity in prepubertal children. Clin. Endocrinol..

[3] Jung M.H., Suh B.K., Ko C.W., Lee K.H., Jin D.K., Yoo H.W., Hwang J.S., Chung W.Y., Han H.S., Prusty V. (2020). Efficacy and Safety Evaluation of Human Growth Hormone Therapy in Patients with Idiopathic Short Stature in Korea—A Randomised Controlled Trial. Eur. Endocrinol..

[4] Finkelstein B.S., Imperiale T.F., Speroff T., Marrero U., Radcliffe D.J., Cuttler L. (2002). Effect of growth hormone therapy on height in children with idiopathic short stature: A meta-analysis. Arch. Pediatr. Adolesc. Med..

[5] Halas J.G., Grimberg A. (2020). Dilemmas of growth hormone treatment for GH deficiency and idiopathic short stature: Defining, distinguishing, and deciding. Minerva Pediatr..

[6] Lanes R., Gonzalez Briceno L.G. (2017). Alternatives in the Treatment of Short Stature. Adv. Pediatr..

[7] Khawaja N., Owaineh H., Batieha A., Frahid O., El-Khateeb M., Ajlouni K.M. (2019). The Effect of Gonadotropin-Releasing Hormone Analogue on Final Adult Height in Children with Idiopathic Short Stature. Med. Princ. Pract..

[8] Mauras N., Ross J.L., Gagliardi P., Yu Y.M., Hossain J., Permuy J., Damaso L., Merinbaum D., Singh R.J., Gaete X. (2016). Randomized Trial of Aromatase Inhibitors, Growth Hormone, or Combination in Pubertal Boys with Idiopathic, Short Stature. J. Clin. Endocrinol. Metab..

[9] Lee B., Kwon C.Y., Jang S. (2022). Comparative effectiveness of East Asian traditional medicine for treatment of idiopathic short stature in children: Systematic review and network meta-analysis. Integr. Med. Res..

[10] Jang S., Lee S.H., Kim Y.J., Lee B.R. (2023). Cost-effectiveness analysis of herbal medicines in children with idiopathic short stature. Medicine.

[11] Lee D., Lee S.H., Song J., Jee H.J., Cha S.H., Chang G.T. (2018). Effects of Astragalus Extract Mixture HT042 on Height Growth in Children with Mild Short Stature: A Multicenter Randomized Controlled Trial. Phytother. Res..

[12] Li Y., Chen X., Liu Z., Yang J. (2022). Chinese herbal medicine for children with idiopathic short stature (ISS): A systematic review and meta-analysis. PLoS ONE.

[13] Renaville R., Hammadi M., Portetelle D. (2002). Role of the somatotropic axis in the mammalian metabolism. Domest. Anim. Endocrinol..

[14] Park P., Cohen P. (2005). Insulin-like growth factor I (IGF-I) measurements in growth hormone (GH) therapy of idiopathic short stature (ISS). Growth Horm. Igf Res..

[15] Goth M.I., Lyons C.E., Canny B.J., Thorner M.O. (1992). Pituitary adenylate cyclase activating polypeptide, growth hormone (GH)-releasing peptide and GH-releasing hormone stimulate GH release through distinct pituitary receptors. Endocrinology.

[16] Yakar S., Rosen C.J., Beamer W.G., Ackert-Bicknell C.L., Wu Y., Liu J.L., Ooi G.T., Setser J., Frystyk J., Boisclair Y.R. (2002). Circulating levels of IGF-1 directly regulate bone growth and density. J. Clin. Investig..

[17] Fang J., Zhang X., Chen X., Wang Z., Zheng S., Cheng Y., Liu S., Hao L. (2023). The role of insulin-like growth factor-1 in bone remodeling: A review. Int. J. Biol. Macromol..

[18] Wang Y., Zhang H., Cao M., Kong L., Ge X. (2019). Analysis of the value and correlation of IGF-1 with GH and IGFBP-3 in the diagnosis of dwarfism. Exp. Ther. Med..

[19] Byers S., Moore A.J., Byard R.W., Fazzalari N.L. (2000). Quantitative histomorphometric analysis of the human growth plate from birth to adolescence. Bone.

[20] Kim H., Fu Y., Hong H.J., Lee S.G., Lee D.S., Kim H.M. (2022). Structural basis for assembly and disassembly of the IGF/IGFBP/ALS ternary complex. Nat. Commun..

[21] MacRae V.E., Farquharson C., Ahmed S.F. (2006). The pathophysiology of the growth plate in juvenile idiopathic arthritis. Rheumatology.

[22] Christians J.K., Hoeflich A., Keightley P.D. (2006). PAPPA2, an enzyme that cleaves an insulin-like growth-factor-binding protein, is a candidate gene for a quantitative trait locus affecting body size in mice. Genetics.

[23] Prein C., Warmbold N., Farkas Z., Schieker M., Aszodi A., Clausen-Schaumann H. (2016). Structural and mechanical properties of the proliferative zone of the developing murine growth plate cartilage assessed by atomic force microscopy. Matrix Biol..

[24] Lampl M., Schoen M. (2017). How long bones grow children: Mechanistic paths to variation in human height growth. Am. J. Hum. Biol..

[25] Shim N.Y., Heo J.S. (2021). Performance of the Polydopamine-Graphene Oxide Composite Substrate in the Osteogenic Differentiation of Mouse Embryonic Stem Cells. Int. J. Mol. Sci..

[26] Lu N., Malemud C.J. (2019). Extracellular Signal-Regulated Kinase: A Regulator of Cell Growth, Inflammation, Chondrocyte and Bone Cell Receptor-Mediated Gene Expression. Int. J. Mol. Sci..

[27] Lazarus J.E., Hegde A., Andrade A.C., Nilsson O., Baron J. (2007). Fibroblast growth factor expression in the postnatal growth plate. Bone.

[28] Faienza M.F., Chiarito M., Brunetti G., D’Amato G. (2021). Growth plate gene involment and isolated short stature. Endocrine.

[29] Funari M.F.A., de Barros J.S., Santana L.S., Lerario A.M., Freire B.L., Homma T.K., Vasques G.A., Mendonca B.B., Nishi M.Y., Jorge A.A.L. (2019). Evaluation of defects in the era of next-generation sequencing. Clin. Genet..

[30] Marouli E., Graff M., Medina-Gomez C., Lo K.S., Wood A.R., Kjaer T.R., Fine R.S., Lu Y., Schurmann C., Highland H.M. (2017). Rare and low-frequency coding variants alter human adult height. Nature.

[31] Iglesias L., Yeh J.K., Castro-Magana M., Aloia J.F. (2011). Effects of growth hormone on bone modeling and remodeling in hypophysectomized young female rats: A bone histomorphometric study. J. Bone Miner. Metab..

[32] Zhang Z.X., Liu Y.K., Pan H., Pan L., Zhang Q., Su H.M., Zhao Q.L., Li H., He C. (2012). The effect of polyethylene glycol recombinant human growth hormone on growth and glucose metabolism in hypophysectomized rats. Growth Horm. Igf Res..

[33] Bright G.M., Fierro-Renoy J.F. (2020). A rationale for the treatment of short stature in children with the combination of recombinant human growth hormone (rhGH) and recombinant human insulin-like growth factor-I (rhIGF-I). Growth Horm. Igf Res..

[34] Sengupta P. (2013). The Laboratory Rat: Relating Its Age With Human’s. Int. J. Prev. Med..

[35] Hansson L.I., Menander-Sellman K., Stenstrom A., Thorngren K.G. (1972). Rate of normal longitudinal bone growth in the rat. Calcif. Tissue Res..

[36] Al Shaikh A., Daftardar H., Alghamdi A.A., Jamjoom M., Awidah S., Ahmed M.E., Soliman A.T. (2020). Effect of growth hormone treatment on children with idiopathic short stature (ISS), idiopathic growth hormone deficiency (IGHD), small for gestational age (SGA) and Turner syndrome (TS) in a tertiary care center. Acta Biomed..

[37] Han S.Y., Kim J.H., Jo E.H., Kim Y.K. (2021). Eleutherococcus sessiliflorus Inhibits Receptor Activator of Nuclear Factor Kappa-B Ligand (RANKL)-Induced Osteoclast Differentiation and Prevents Ovariectomy (OVX)-Induced Bone Loss. Molecules.

[38] Qian J.Y., Bai Y.Y., Tang J., Chen W. (2015). Antioxidation and alpha-glucosidase inhibitory activities of barley polysaccharides modified with sulfation. Lwt-Food Sci. Technol..

[39] Lee D., Lee S.H., Cho N., Kim Y.S., Song J., Kim H. (2019). Effects of Eleutherococcus Extract Mixture on Endochondral Bone Formation in Rats. Int. J. Mol. Sci..

[40] Kim M.Y., Park Y., Pandit N.R., Kim J., Song M., Park J., Choi H.Y., Kim H. (2010). The herbal formula HT042 induces longitudinal bone growth in adolescent female rats. J. Med. Food.

[41] Lee D., Kim B.H., Lee S.H., Cho W.Y., Kim Y.S., Kim H. (2021). Effects of Astragalus Extract Mixture HT042 on Circulating IGF-1 Level and Growth Hormone Axis in Rats. Children.

[42] Kim Y.B., Kang E.J., Noh J.R., An J.P., Park J.T., Oh W.K., Kim Y.H., Lee C.H. (2023). Humulus japonicus ameliorates irritant contact dermatitis by suppressing NF-kappaB p65-dependent inflammatory responses in mice. Exp. Ther. Med..

[43] Kim O.K., Yun J.M., Lee M., Park S.J., Kim D., Oh D.H., Kim H.S., Kim G.Y. (2020). A Mixture of Humulus japonicus Increases Longitudinal Bone Growth Rate in Sprague Dawley Rats. Nutrients.

[44] Kim O.K., Yun J.M., Lee M., Park S.J., Kim D., Oh D.H., Kim H.S., Lee J. (2021). Effects of a Mixture of Humulus japonicus on Longitudinal Bone Growth in Hypophysectomized Rats. J. Med. Food.

[45] Lee S., Kwon D.H., Kim J.Y., Kim Y., Cho S.H., Jung I.C. (2021). Efficacy of Yukmijihwang-tang on symptoms of Alzheimer disease: A protocol for systematic review and meta-analysis. Medicine.

[46] Kang J.Y., Lee J.S., Seol I.C., Kim Y.S., Park M.S., Yoo H.R. (2022). Pharmacological Effects of Gami-Yukmijihwang-Tang on the Lipopolysaccharide-Induced Hippocampus Oxidation and Inflammation via Regulation of Sirt6. Pharmaceuticals.

[47] Cho S.M., Lee S.H., Lee D., Lee J.H., Chang G.T., Kim H., Lee J.Y. (2017). The Korean herbal formulation Yukmijihwangtang stimulates longitudinal bone growth in animal models. BMC Complem. Altern. Med..

[48] Jang S., Lee B. (2022). Clinical Practice Pattern of Korean Medicine Doctors in Idiopathic Short Stature Treatment: A Survey Study. Evid. Based Complement. Alternat. Med..

[49] Leem K., Park S.Y., Lee D.H., Boo Y.M., Cho K.H., Lim J., Jeon H., Park H.J., Chung J.H., Kim H. (2003). Effects of Jaoga-Yukmiwon (R), a Korean herbal medicine, on chondrocyte proliferation and longitudinal bone growth in adolescent male rats. Phytother. Res..

[50] Wang X.Z., Wang T., Wang Y.Z., Li X., Chen Q., Wang Y., Zhang X.Y., Wang H.X., Zhao H.J., Mou Y. (2022). Research progress on classical traditional Chinese medicine Taohong Siwu decoction in the treatment of coronary heart disease. Biomed. Pharmacother..

[51] Lee D., Lee S.H., Lee M., Lee S.H., Shin Y.J., Lee J.Y., Kim H., Kim Y.S., Song J. (2019). Effects of Siwu decoction on chondrocyte proliferation of growth plate in adolescent rats. J. Ethnopharmacol..

[52] Hwang S.Y., Lee S.H., Lee Y.S., Han S.H., Song B.H., Reddy C.S., Kim Y.B., Park S.U. (2020). Molecular Characterization of Terpenoid Biosynthetic Genes and Terpenoid Accumulation in Turczaninow. Horticulturae.

[53] Li H., Liu Q.S., Zhou X.R., Sui H., Fu X.Y. (2021). Phlomoides umbrosa (Turcz.) Kamelin & Makhm: A review of its traditional uses, botany, phytochemistry, pharmacology and clinical research. J. Ethnopharmacol..

[54] Lee D., Kim Y.S., Song J., Kim H.S., Lee H.J., Guo H., Kim H. (2016). Effects of Phlomis umbrosa Root on Longitudinal Bone Growth Rate in Adolescent Female Rats. Molecules.

[55] Aoyama S., Yamamoto Y. (2007). Antioxidant activity and flavonoid content of Welsh onion (*Allium fistulosum*) and the effect of thermal treatment. Food Sci. Technol. Res..

[56] Ryuk J.A., Kim H.J., Hwang J.T., Ko B.S. (2021). Effect of Allium fistulosum Extracts on the Stimulation of Longitudinal Bone Growth in Animal Modeling Diet-Induced Calcium and Vitamin D Deficiencies. Appl. Sci..

[57] Xie H.L., Shi X.L., Zhao D.X., Wang B.Z., Jin Y.R., Li X.W. (2023). A review: The structures and bioactivities of steroidal saponins from macrostemon Bulbus. Phytochem. Lett..

[58] Wang H.X., Zheng Q., Dong A.J., Wang J.C., Si J.Y. (2023). Chemical Constituents, Biological Activities, and Proposed Biosynthetic Pathways of Steroidal Saponins from Healthy Nutritious Vegetable-. Nutrients.

[59] Kim H.J., Lee S.H., Lee S.H., Lee J., Kim H., Chang G.T., Lee D. (2020). Longitudinal Bone Growth Stimulating Effect of Allium macrostemon in Adolescent Female Rats. Molecules.

[60] Yue J.J., Zhang S.L., Zheng B., Raza F., Luo Z.H., Li X.H., Zhang Y.Y., Nie Q., Qiu M.F. (2021). Efficacy and Mechanism of Active Fractions in Fruit of Lour. for Gastric Cancer. J. Cancer.

[61] Lee S.H., Kim J.Y., Kim H., Park S.K., Kim C.Y., Chung S.Y., Chang G.T. (2012). Amomum villosum induces longitudinal bone growth in adolescent female rats. J. Tradit. Chin. Med..

[62] Xing Y.F., He D., Wang Y., Zeng W., Zhang C., Lu Y., Su N., Kong Y.H., Xing X.H. (2019). Chemical constituents, biological functions and pharmacological effects for comprehensive utilization of Oliver. Food Sci. Hum. Well.

[63] Ha H., Ho J.Y., Shin S., Kim H., Koo S., Kim I.H., Kim C. (2003). Effects of eucommiae cortex on osteoblast-like cell proliferation and osteoclast inhibition. Arch. Pharm. Res..

[64] Kim J.Y., Lee J.I., Song M., Lee D., Song J., Kim S.Y., Park J., Choi H.Y., Kim H. (2015). Effects of Eucommia ulmoides extract on longitudinal bone growth rate in adolescent female rats. Phytother. Res..

[65] Kim A., Im M., Yim N.H., Jung Y.P., Ma J.Y. (2013). Aqueous Extract of Bambusae Caulis in Taeniam Inhibits PMA-Induced Tumor Cell Invasion and Pulmonary Metastasis: Suppression of NF-kappa B Activation through ROS Signaling. PLoS ONE.

[66] Kim W., Lim D., Kim J. (2018). p-Coumaric Acid, a Major Active Compound of Bambusae Caulis in Taeniam, Suppresses Cigarette Smoke-Induced Pulmonary Inflammation. Am. J. Chinese Med..

[67] Chung Y.H., Lee D.Y., Lee H.S., Hong S.A., Park E.S., Nam Y., Kim H.C., Lee S.J., Sohn U.D., Kim H. (2016). Effects of Aqueous Extract of Phyllostachyos Caulis in Taeniam on Longitudinal Bone Growth in Adolescent Rats. Planta Med..

[68] Balazova L., Kurhajec S., Kello M., Bedlovicova Z., Zigova M., Petrovova E., Benova K., Mojzis J., Eftimova J. (2022). Antiproliferative Effect of Phellodendron amurense Rupr. Based on Angiogenesis. Life.

[69] Okuda T., Jo R., Tsutsumi K., Watai D., Ishihara C., Yama K., Aita Y., Inokuchi T., Kimura M., Chikazawa T. (2023). An in vitro study of the effects of Phellodendron bark extract and berberine chloride on periodontal pathogenic bacteria in the oral microbiome. J. Oral Biosci..

[70] Lee S.H., Lee H.J., Lee S.H., Kim Y.S., Lee D., Chun J., Lee J.Y., Kim H., Chang G.T. (2018). Effects of Huang Bai (Phellodendri Cortex) on bone growth and pubertal development in adolescent female rats. Chin. Med..

[71] Kim H.K., Kim M.G., Leem K.H. (2016). Comparison of the Effect of Velvet Antler from Different Sections on Longitudinal Bone Growth of Adolescent Rats. Evid.-Based Compl. Alt..

[72] Zhang S.Y., Zhao G.X., Suo S.K., Wang Y.M., Chi C.F., Wang B. (2021). Purification, Identification, Activity Evaluation, and Stability of Antioxidant Peptides from Alcalase Hydrolysate of Antarctic Krill (*Euphausia superba*) Proteins. Mar. Drugs.

[73] Dai Y.F., Li Z., Fu M., Li Y.Q., Xue C.H., Wang J.F. (2021). Peptides from Euphausia superba Promote Longitudinal Bone Growth by Accelerating Growth Plate Chondrocyte Proliferation and Hypertrophy. Curr. Pharm. Biotechnol..

[74] Jiang S., Qu X.J., Liu S.P., Wei J., Yi X.X., Liu Y.H., Gao C.H. (2023). Proteomic Identification of Plasma Components in Tachypleus tridentatus and Their Effects on the Longitudinal Bone Growth Rate in Rats. Mar. Drugs.

[75] Bajzelj B., Laguzzi F., Roos E. (2021). The role of fats in the transition to sustainable diets. Lancet Planet Health.

[76] Koren N., Simsa-Maziel S., Shahar R., Schwartz B., Monsonego-Ornan E. (2014). Exposure to omega-3 fatty acids at early age accelerate bone growth and improve bone quality. J. Nutr. Biochem..

[77] Zbinden-Foncea H., Castro-Sepulveda M., Fuentes J., Speisky H. (2022). Effect of Epicatechin on Skeletal Muscle. Curr. Med. Chem..

[78] Kaur J., Kaur R. (2022). Coumaric Acid: A Naturally Occurring Chemical with Potential Therapeutic Applications. Curr. Org. Chem..

[79] Lee J.H., Chung Y.H., Kim H.H., Bang J.S., Jung T.W., Park T., Park J., Kim U., Lee S.H., Jeong J.H. (2018). p-Coumaric acid stimulates longitudinal bone growth through increasing the serum production and expression levels of insulin-like growth factor 1 in rats. Biochem. Bioph. Res. Commun..

[80] Wu X.G., Zhang T., Yang H.J., Yue Y., Kim M.J., Li C., Cheong S.I., Jang D.J., Park S. (2023). Promotion of longitudinal bone growth by the intake of oat and green onion root water extracts in weaning rats through stimulating growth hormone secretion and elevating gut microbiota related to nervous system-related pathway. J. Funct. Foods.

[81] Choudhary D., Pandey A., Adhikary S., Ahmad N., Bhatia C., Bhambhani S., Trivedi P.K., Trivedi R. (2016). Genetically engineered flavonol enriched tomato fruit modulates chondrogenesis to increase bone length in growing animals. Sci. Rep..

